# Development and Validation of a Machine Learning-Based Risk Assessment Tool for In-Hospital Mortality in Elderly Patients with Postoperative Hypoxemia Following Non-Cardiac Surgery

**DOI:** 10.3390/jcm15124725

**Published:** 2026-06-18

**Authors:** Yuchen Zhou, Xinhe Zhou, Xiaozhu Liu, Chenghui Zhou, Yang Liu

**Affiliations:** 1 Department of Surgical Intensive Care Unit, Beijing Shijitan Hospital, Capital Medical University, No. 10, Tieyi Road, Haidian District, Beijing 100038, China; zhouyuchen@bjsjth.cn; 2 Capital Medical University, No. 10, Right Out West First Street, Fengtai District, Beijing 100069, China; 17778022330@163.com (X.Z.); xiaozhuliu2021@163.com (X.L.); 3 Department of Anesthesiology, Beijing Luhe Hospital, Capital Medical University, No. 82, Xinhua South Road, Tongzhou District, Beijing 101199, China; chenghuizhou@vip.163.com; 4 Department of Critical Care Medicine, Beijing Luhe Hospital, Capital Medical University, No. 82, Xinhua South Road, Tongzhou District, Beijing 101199, China

**Keywords:** postoperative hypoxemia, non-cardiac surgery, mortality prediction, machine learning

## Abstract

**Background/Objectives:** Postoperative hypoxemia is a frequent complication after non-cardiac surgery and is correlated with elevated mortality rates in elderly patients. However, a dedicated predictive tool for mortality in this specific patient subgroup remains unavailable. To construct and validate a machine learning (ML) model for predicting in-hospital mortality among elderly adults who develop hypoxemia after non-cardiac surgery. **Methods:** Data for this retrospective cohort study were obtained from the Medical Information Mart for Intensive Care IV (MIMIC-IV) database. The study encompassed patients aged 65 years or older who exhibited hypoxemia, defined as a PaO_2_/FiO_2_ ratio below 300 mmHg, within the initial 48 h of intensive care unit (ICU) stay. LASSO (Least Absolute Shrinkage and Selection Operator) regression was applied for feature selection, after which six distinct machine learning models and five conventional scoring systems were constructed and evaluated. SHapley Additive exPlanations (SHAP) was employed to improve model interpretability. **Results:** Out of 6051 eligible patients, 1838 (30.4%) succumbed during hospitalization. The XGBoost algorithm demonstrated superior predictive capability, achieving an area under the curve (AUC) of 0.794, along with a specificity of 0.917, accuracy of 0.769, and positive predictive value of 0.693. Critical predictors identified included administration of vasopressors, advanced age, and the PaO_2_/FiO_2_ ratio. **Conclusions:** The Extreme Gradient Boosting (XGBoost)-driven ML model provides accurate prediction of in-hospital mortality in elderly patients with postoperative hypoxemia after non-cardiac surgery, presenting a valuable instrument for early risk evaluation and potential intervention.

## 1. Introduction

Postoperative hypoxemia represents a common clinical challenge in patients recovering from non-cardiac surgical procedures, with reported incidence rates varying between 12% and 50 [1,2,3,4]. While many instances are mild and self-limiting, persistent hypoxemic episodes are linked to adverse clinical outcomes, such as extended duration of respiratory support, prolonged hospitalization, and escalated healthcare expenditures [5,6,7]. The underlying pathophysiological mechanisms often involve atelectasis, ventilation-perfusion mismatch, or pulmonary edema [4]. Elderly patients are at higher risk of postoperative hypoxemia due to their respiratory muscle weakness and reduced lung reserve. Although early detection of hypoxemia can inform tailored therapeutic strategies—ranging from conventional oxygen supplementation to high-flow nasal cannula (HFNC) therapy and noninvasive or invasive ventilatory support—moderate to severe cases are associated with a higher mortality, compared with younger patients.

Current approaches to estimating mortality risk in patients with postoperative hypoxemia largely depend on preoperative comorbidities, surgical characteristics, and operative duration. While previous efforts have been made to construct predictive models for postoperative complications [8,9,10], a specialized scoring system for predicting mortality specifically in non-cardiac surgery patients with postoperative hypoxemia has not been established, especially for elderly patients.

Consequently, the principal aim of this investigation was to devise a novel risk assessment tool to accurately forecast mortality in this patient population.

## 2. Methods

### 2.1. Study Design and Data Sources

The present retrospective cohort study employed electronic health records from the Medical Information Mart for Intensive Care IV (MIMIC-IV) database [11]. This resource encompasses clinical data collected from critically ill individuals receiving care in the intensive care unit (ICU) at Beth Israel Deaconess Medical Center (BIDMC), Boston, Massachusetts, during the period from 2008 to 2022. Author CHZH acquired authorized access to the database and was tasked with data extraction (certification number: [144453599]). Approval for database access was granted by the Massachusetts Institute of Technology Affiliates (ID: 61043182). The MIMIC database was established following ethical approval from the Institutional Review Boards of Beth Israel Deaconess Medical Center (2001-P-001699/14) and the Massachusetts Institute of Technology (No. 0403000206). Both boards waived the requirement for individual informed consent and approved the data-sharing initiative. We conducted this study in compliance with the TRIPOD (Transparent Reporting of a multivariable prediction model for Individual Prognosis Or Diagnosis) guidelines [12]. Considering the retrospective observational nature of our research, informed consent was waived by the ethics committee. All procedures were performed in strict adherence to medical ethical principles.

### 2.2. Study Population

Participants in this study were adult patients aged 65 years or older who received non-cardiac surgery and subsequently developed hypoxemia during the initial 48 h after ICU admission. Hypoxemia was defined as a PaO_2_/FiO_2_ ratio less than 300 mmHg. All non-cardiac surgery patients within the MIMIC-IV dataset were considered eligible. Exclusion criteria consisted of: absence of postoperative hypoxemia or pre-existing hypoxemia prior to surgery, missing PaO_2_/FiO_2_ data, or more than 30% missing data for any key indicator.

A wide range of predictor variables were retrieved from electronic health records, encompassing demographic characteristics such as age, gender, body mass index (BMI), and race/ethnicity, comorbid conditions (myocardial infarction, congestive heart failure, peripheral vascular disease, cerebrovascular disease, dementia, chronic pulmonary disease, rheumatic disease, peptic ulcer disease, mild liver disease, diabetes, paraplegia, renal disease, malignant cancer, severe liver disease, metastatic solid tumor), vital signs (heart rate, respiratory rate), laboratory findings (serum creatinine, blood urea nitrogen [BUN]), severity scores (Acute Physiology Score III [APSIII], Sequential Organ Failure Assessment [SOFA], Logistic Organ Dysfunction System [LODS], Oxford Acute Severity of Illness Score [OASIS], Simplified Acute Physiology Score III [SAPSIII]), along with surgical type and treatment-related variables (continuous renal replacement therapy or vasopressor use). Only variables documented in over 70% of the cohort were retained. Structured Query Language (SQL) was employed for data extraction through PostgreSQL (version 10.0), and subsequent data cleaning and organization were performed using the R software (version 4.4.2) environment.

### 2.3. Feature Selection

During feature selection, fifty-seven variables were initially considered based on the pathophysiology, clinical presentation, and management of hypoxemia, in conjunction with the study’s inclusion and exclusion criteria. Subsequently, these variables were obtained from the Electronic Health Record (EHR) system to support modeling and screening. The primary outcome measure was in-hospital mortality, established based on patient survival status at the time of discharge (survived or deceased).

In order to reduce the possible effects of missing data on model performance, any variables with a missing rate surpassing 30% were excluded. For the residual missing values, the method of multiple imputation by chained equations (MICE) was utilized, recognized for its robustness and accuracy in handling datasets containing moderate amounts of missing data [13].

We implemented Least Absolute Shrinkage and Selection Operator (LASSO) regression for additional variable refinement. The technique utilizes a regularization penalty to reduce coefficients of less important variables toward zero, thereby maintaining predictors with more significant associations. A predictor–outcome matrix was constructed, and ten-fold cross-validation with minimal prediction error was employed to optimize the LASSO model, identifying the ideal lambda (λ) value for selecting the most pertinent predictors for in-hospital mortality in the training set. This approach effectively reduces redundancy and preserves clinically significant features linked to the outcome, enhancing predictive accuracy and diminishing overfitting risk. Individuals lacking discharge status documentation were removed from the final analytical cohort. In the end, twenty-five features were selected and integrated into a consolidated dataset for later model development and validation.

### 2.4. Model Development, Validation, and Interpretation

Through stratified random sampling, the dataset was split into two components: a training set (70%) for model development and a test set (30%) for independent validation. The test set was strictly reserved for assessing model generalizability, ensuring unbiased evaluation.

We employed six machine learning (ML) algorithms that are widely used in practice to create predictive models for in-hospital mortality: Radial Support Vector Machine (RSVM), Extreme Gradient Boosting (XGBoost), Light Gradient Boosting Machine (LightGBM), Multi-layer Perceptron (MLP), Random Forest (RF), and Decision Tree (DT). Hyperparameter tuning for all models was performed using ten-fold cross-validation integrated with Grid Search. The test set remained unused during the tuning process to avoid information leakage and inflated performance estimates.

Post-training evaluation of predictive performance utilized various metrics, including the Receiver Operating Characteristic (ROC) curve with its Area Under the Curve (AUC), Sensitivity, Specificity, Accuracy, F1-score, Positive Predictive Value (PPV), and Negative Predictive Value (NPV). To assess prediction reliability, calibration curves were generated; furthermore, Decision Curve Analysis (DCA) was conducted to measure clinical net benefit at varying threshold probabilities. The primary objective was to identify the top-performing model, which underwent subsequent re-evaluation on the validation set to confirm both stability and generalizability.

To augment interpretability, the SHapley Additive exPlanations (SHAP) framework was implemented to elucidate the predictions of the top-performing model. The SHAP summary plot illustrated the overall influence of each feature on model output, while a bar chart ranked variables by importance. SHAP dependence plots provided localized explanations, demonstrating how variations in individual feature values affected predictive outcomes, thereby facilitating clinical comprehension of the model’s decision-making process.

### 2.5. Statistical Analysis

Continuous variable normality was tested using the Shapiro–Wilk procedure. Data with normal distribution were summarized as mean ± standard deviation and compared via independent samples *t*-tests. Non-normal data were reported as median with interquartile range [M (P25, P75)] and analyzed using the Wilcoxon rank-sum test. Categorical data were expressed as frequencies and percentages [*n* (%)], with statistical comparisons made using chi-square tests or Fisher’s exact tests according to data characteristics. Predictive modeling incorporated the six ML algorithms described. All analyses were performed in R (version 4.4.3), with a *p*-value < 0.05 deemed statistically significant.

## 3. Results

### 3.1. Clinical Characteristics and Univariate Analysis

**Figure 1:**
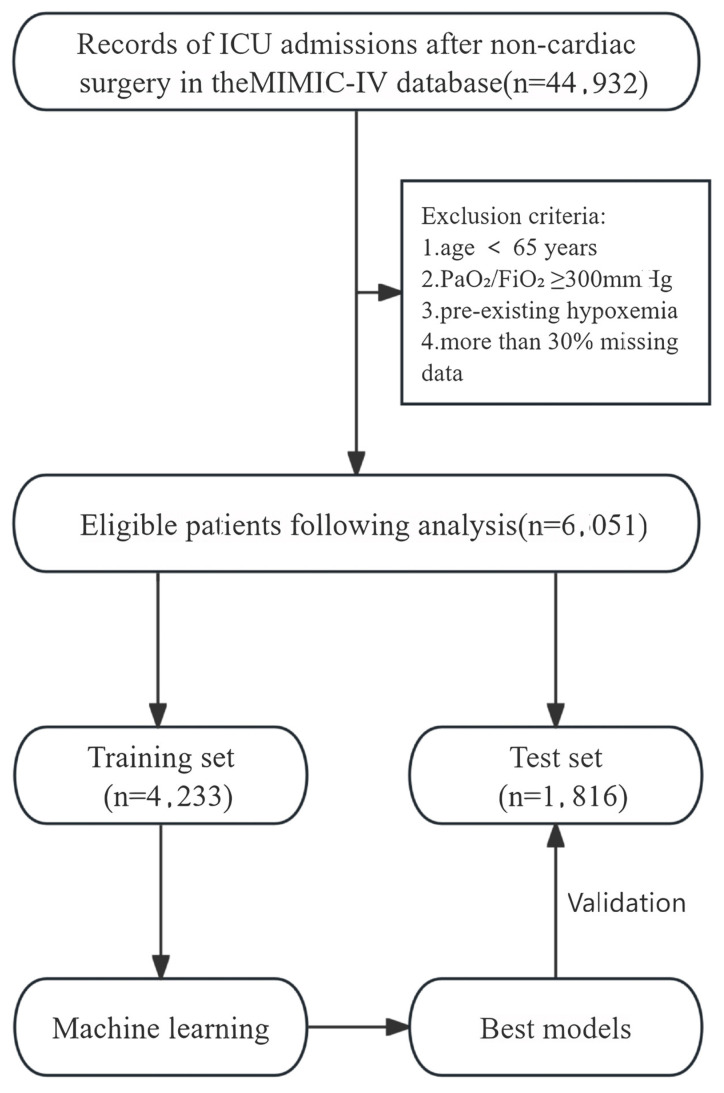
Patient screening flow from the MIMIC database.

As delineated in Figure 1, the study included 6051 patients from the MIMIC-IV database, among whom 1838 (30.4%) died following ICU transfer. The cohort was divided into a training set (*n* = 4233) and a test set (*n* = 1816), with both subsets exhibiting a mortality rate of 30.4%. Baseline clinical, laboratory, and procedural characteristics for both cohorts are detailed in Table 1. In the training set, nearly all parameters demonstrated statistically significant differences between survivors and non-survivors, except for gender, chloride, hemoglobin, congestive heart failure, rheumatic disease, peptic ulcer disease, diabetes, malignant cancer, infective endocarditis (IE), pulmonary artery hypertension (PAH), atrial fibrillation (AF), and acquired immune deficiency syndrome (AIDS) (*p* > 0.05). Similarly, in the test set, no significant differences were observed for gender, diastolic blood pressure (Dbp), calcium, glucose, sodium, hemoglobin, congestive heart failure, dementia, rheumatic disease, peptic ulcer disease, diabetes, renal disease, malignant cancer, severe liver disease, IE, PAH, AF, and AIDS at enrollment (*p* > 0.05).

### 3.2. Feature Determination and Model Construction

**Figure 2:**
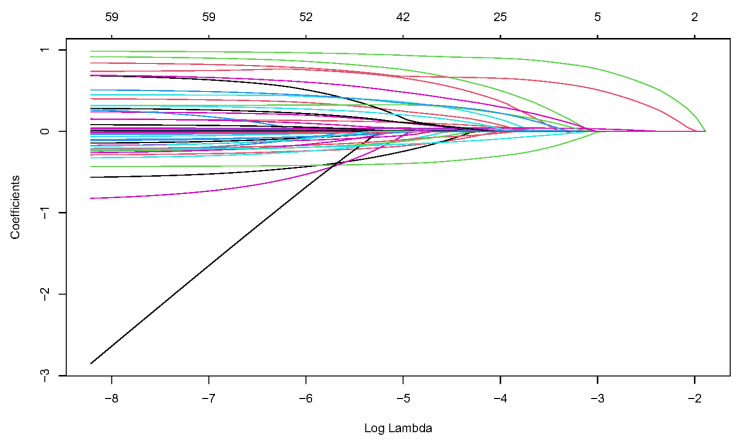
Variation characteristics of variable coefficients.

**Figure 3:**
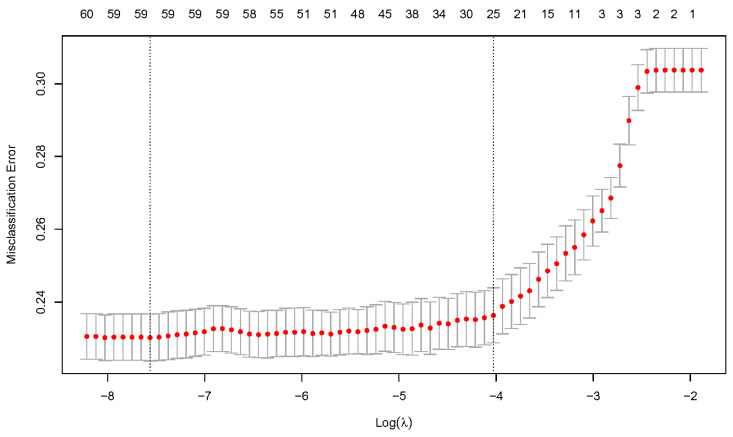
The process of selecting the optimal value of the parameter λ in the lasso regression model.

LASSO regression analysis was performed with mortality as the dependent variable, incorporating the initial fifty-seven variables. Using an optimal shrinkage parameter lambda (λ) value of 0.0021 (Figure 2 and Figure 3), twenty-five independent predictors were retained for the final risk model. These included: PaO_2_/FiO_2_ ratio, ethnicity, age, weight, peripheral vascular disease, cerebrovascular disease, mild liver disease, paraplegia, metastatic solid tumor, hyperlipidemia, hypertension, heart rate, respiratory rate, temperature, anion gap, blood BUN, alkaline phosphatase, prothrombin time, white blood cell count, magnesium level, and administration of dopamine, dobutamine, epinephrine, norepinephrine, and vasopressin.

**Figure 4:**
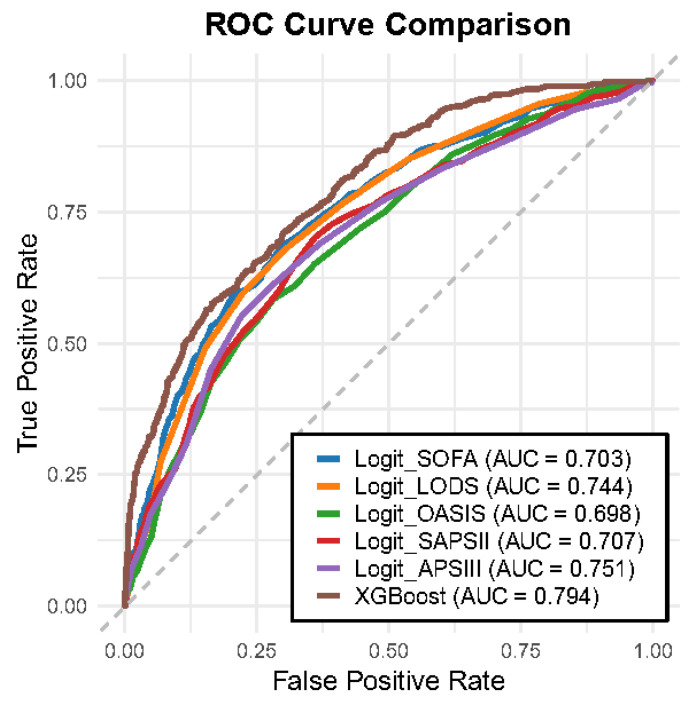
ROC curves for the six machine learning models. DT: Decision Tree; LightGBM: Light Gradient Boosting Machine; MLP: Multi-layer Perceptron; RF: Random Forest; RSVM: Radial Support Vector Machine; XGBoost: Extreme Gradient Boosting; ROC: receiver operating characteristic; AUC: area under the curve.

**Figure 5:**
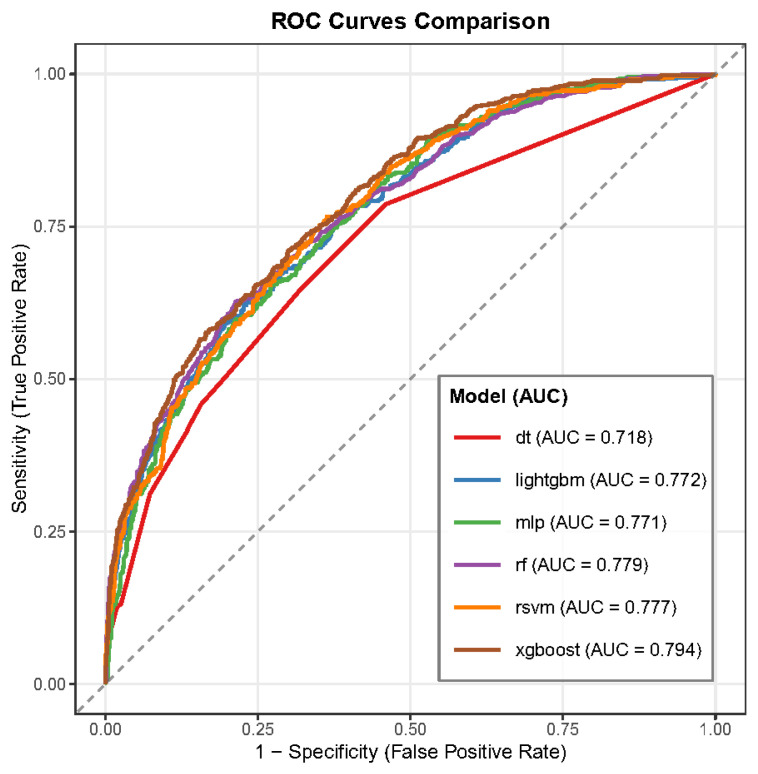
ROC curves between XGBoost and other five scoring systems. SOFA: Sequential Organ Failure Assessment; LODS: Logistic Organ Dysfunction System; OASIS: Oxford Acute Severity of Illness Score; APSIII: Acute Physiology Score III; SAPSIII: Simplified Acute Physiology Score III; XGBoost: Extreme Gradient Boosting; ROC: receiver operating characteristic; AUC: area under the curve.

**Figure 6:**
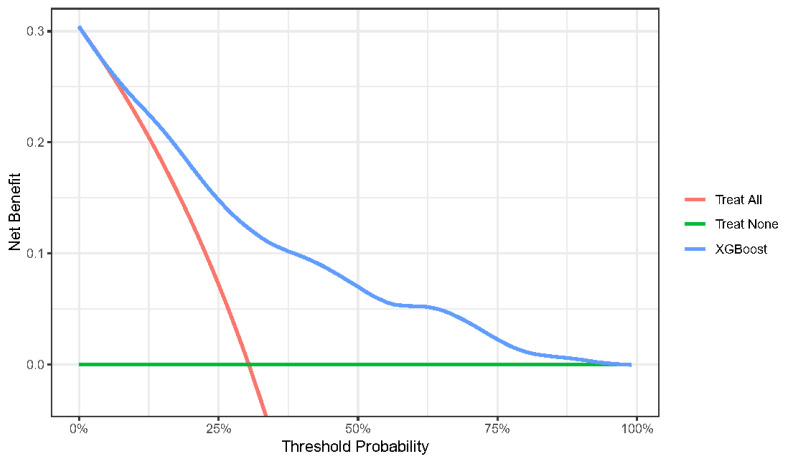
Decision curve analysis of the XGBoost model.

**Figure 7:**
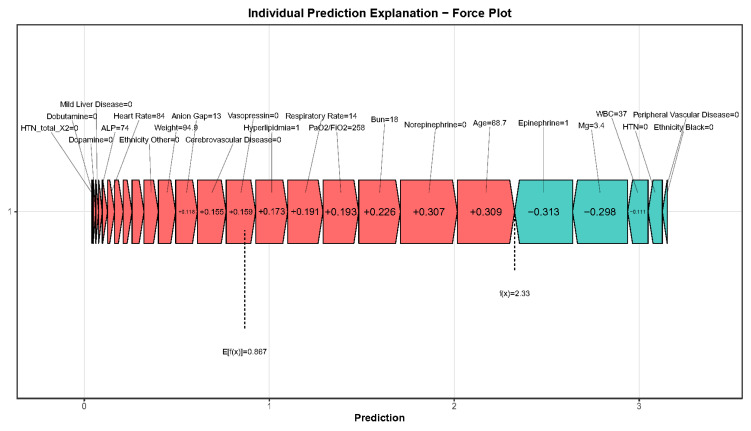
Calibration curve analysis of the XGBoost model.

Six ML models and five scoring systems were developed to predict mortality in the defined patient population. Model performance, evaluated via AUC metrics, is visualized in the ROC curves presented in Figure 4 and Figure 5. The XGBoost model emerged as the top performer (AUC = 0.794), succeeded by Random Forest (AUC = 0.779), RSVM (AUC = 0.777), LightGBM (AUC = 0.772), MLP (AUC = 0.771), APSIII (AUC = 0.751), LODS (AUC = 0.744), Decision Tree (AUC = 0.718), SAPSII (AUC = 0.707), SOFA (AUC = 0.703), and OASIS (AUC = 0.698). Detailed performance metrics for the six ML models are provided in Table 2. The XGBoost model achieved the highest predictive accuracy, specificity (0.917), overall accuracy (0.769), and PPV (0.693). DCA indicated superior clinical applicability for the XGBoost model, and calibration curves confirmed its prediction accuracy (Figure 6 and Figure 7). Collectively, these results establish XGBoost as the optimal model for this study, offering high accuracy and favorable clinical utility.

### 3.3. Feature Importance in the XGBoost Model

**Figure 8:**
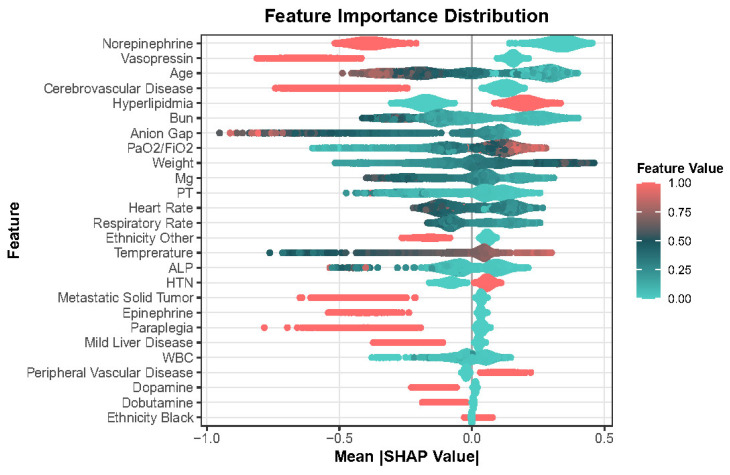
XGBoost model explanation by the SHAP method. BUN: blood urea nitrogen; Mg: magnesium; PT: prothrombin time; ALP: alkaline phosphatase; HTN: hypertension; WBC: white blood cell; SHAP: SHapley Additive exPlanations.

**Figure 9:**
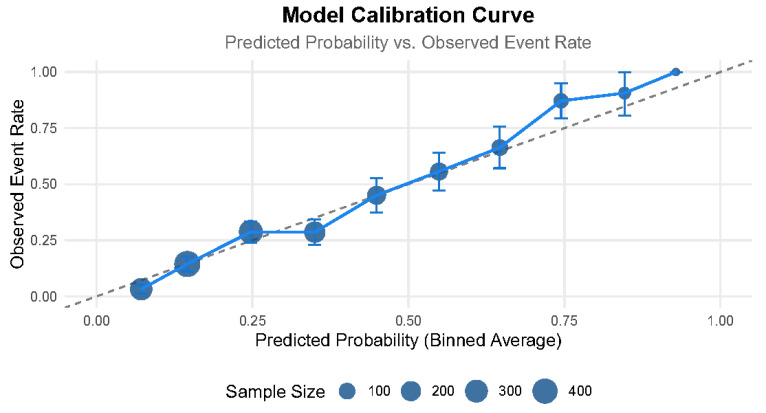
Force plot for one died patient.

**Figure 10:**
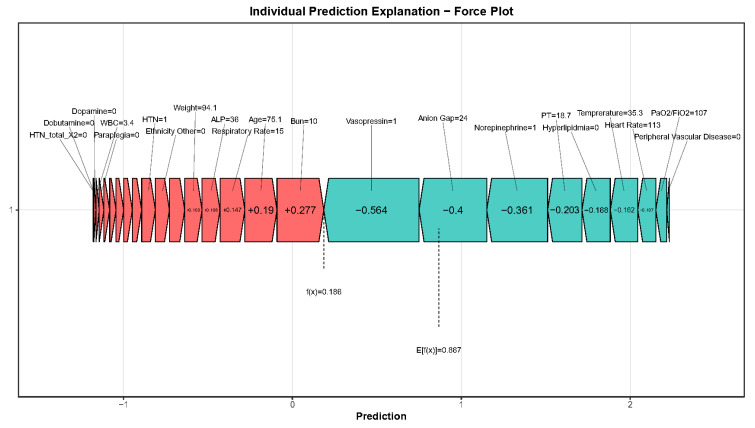
Force plot for one survived patient.

SHAP analysis was employed to quantify the contribution of each variable within the XGBoost model. Figure 8 displays the summary plot of all features, where each point represents a patient’s attribution for mortality risk; red dots signify lower feature values associated with lower risk, while blue dots indicate higher values linked to elevated risk. For illustrative purposes, two representative cases are provided: one patient who died, exhibiting a high SHAP prediction score (2.33) (Figure 9), and another who survived, with a low SHAP score (0.186) (Figure 10), demonstrating the model’s interpretability at an individual level.

### 3.4. Clinical Application Platform

The final XGBoost prediction model was converted into a web-based application to support clinical translation. Through this system, medical practitioners can input individual patient data and receive real-time mortality risk estimates. The tool is accessible in English at https://zhouxinhe.shinyapps.io/XGBoost_predict/ (accessed on 12 March 2006) and in Chinese at https://zhouxinhe.shinyapps.io/XGBoostCN/ (accessed on 12 March 2006).

## 4. Discussion

Postoperative hypoxemia is a potentially severe yet often underrecognized complication. Leveraging a large-scale retrospective dataset, this study developed and validated multiple ML models to predict mortality risk and identify high-risk individuals among elderly patients with postoperative hypoxemia after non-cardiac surgery. The goal was to furnish a more precise predictive instrument for clinical practice, offering novel perspectives for personalized treatment and risk evaluation. Our findings indicate that the XGBoost algorithm delivered robust performance, characterized by strong discriminative ability and calibration, and demonstrated considerable net benefit in a clinical context.

Patients undergoing cardiac surgery typically face a higher risk of postoperative hypoxemia compared to non-cardiac surgery patients [14], attributable to factors like cardiopulmonary bypass, cardiogenic pulmonary edema, and direct lung injury [15,16]. Consequently, hypoxemia following non-cardiac surgery may be overlooked. Several studies have established postoperative hypoxemia as an independent risk factor for mortality [17,18,19]. Recent attempts to build mortality prediction models include a single retrospective study applying traditional logistic regression, which identified an association between the frequency of postoperative hypoxemia events and one-year mortality after adjustment for confounders. Independent predictors in that study included advanced age and other variables [20], indicated that incidence of mortality in the elderly patients with postoperative hypoxemia was higher, and the construction of a mortality prediction model is an important research area for clinicians.

ML models offer distinct advantages through their flexibility in managing incomplete or inconsistent data, thereby reducing prediction biases associated with missing or heterogeneous data [21]. In recent years, ML has exhibited considerable promise in disease prediction and risk assessment [22,23,24,25]. Our study compared six ML algorithms and five conventional scoring systems for mortality prediction, revealing that XGBoost, a gradient boosting decision tree method, significantly surpassed other models. XGBoost-based prediction frameworks have gained widespread traction in healthcare, demonstrating efficacy in domains such as septicemia [26], cardiovascular diseases [27], and kidney injury [28]. This model not only discerns individual variable contributions but also integrates diverse features to yield more precise mortality forecasts. Its built-in regularization controls model complexity to prevent overfitting, while parallel computing ensures computational efficiency—particularly beneficial for large datasets and complex feature interactions. Our XGBoost model achieved an AUC of 0.794, which indicates acceptable discriminative ability according to established interpretation frameworks. However, we acknowledge that the resulting sensitivity of 0.43 falls below optimal levels for mortality prediction, where identifying high-risk patients is paramount. This apparent discrepancy between high AUC and low sensitivity highlights the importance of threshold calibration in clinical applications. As illustrated in ROC analysis, adjusting the classification threshold allows optimization based on specific clinical priorities—for mortality prediction models, lowering the threshold to improve sensitivity (reducing false negatives) is often preferred even at the cost of reduced specificity (increasing false positives). Moreover, the automated and scalable nature of such models renders them highly suitable for clinical environments.

Determining variable importance is crucial yet challenging in both data science and clinical applications. Our study employed SHAP analysis to quantitatively assess each feature’s impact on model predictions. Results indicated that vasopressor use (e.g., norepinephrine, vasopressin) constituted the most influential predictors of mortality risk, with markedly higher mean SHAP values than other features. Yet, it is critical to recognize that vasopressor administration may reflect underlying disease severity rather than being an independent causal determinant of poor outcomes. This phenomenon, known as ‘confounding by indication,’ occurs when both treatment assignment and outcome are influenced by unmeasured severity indicators. Advanced age and age-associated comorbidities also featured prominently, followed by the PaO_2_/FiO_2_ ratio. The human body can be analogized to a complex system wherein the respiratory and circulatory systems serve as the core engine sustaining life. Established critical illness severity scores like SOFA and APACHE II similarly prioritize circulatory and respiratory parameters. A recently proposed hypoxia-age-shock index has shown promising predictive utility for outcomes in COVID-19 patients (AUC = 0.773) [29]. Hypoxia can precipitate reduced oxygen delivery, potentially culminating in Multiple Organ Dysfunction Syndrome (MODS) and elevated lactate levels. It frequently coincides with Acute Kidney Injury (AKI), exacerbating retention of acidic metabolites. Concurrently, hypoxic liver impairment hampers lactate clearance, promoting its accumulation. These pathways can aggravate metabolic acidosis, for which an elevated anion gap serves as a key diagnostic indicator. Prior research has corroborated associations between increased serum anion gap levels and mortality risk in patients with ARDS [30] or gastrointestinal bleeding [31], aligning with our findings. Several retrospective studies in COVID-19 patients have also identified BUN as a paramount predictor of mortality [32,33,34,35]. Preclinical and clinical investigations have reported neuroprotective properties for magnesium sulfate [36,37] and improved in-hospital outcomes in hypoxic–ischemic encephalopathy [38,39]. Additionally, serum magnesium levels are significantly lower in patients with chronic obstructive pulmonary disease (COPD) compared to healthy controls and inversely correlate with exacerbation frequency [40]. While SHAP analysis improved model interpretability by quantifying the contribution of each feature to predictions, it is important to recognize that SHAP values do not imply causality; they merely provide insight into how the models consider various inputs with respect to the output. Our study describes correlational rather than causal relationships between predictors and mortality. Future investigations combining machine learning with causal inference methods could help distinguish risk markers from modifiable risk factors.

Our study has several limitations. First, our study population consisted exclusively of elderly patients (age ≥ 65 years) admitted to the ICU following surgery. Consequently, the applicability of this model to postoperative hypoxemic patients managed in non-critical care settings, such as step-down units, telemetry wards, or general hospital wards, requires further investigation. Second, several important predictors were not incorporated into our model, including intraoperative ventilation strategies, oxygen administration protocols, anesthetic techniques, duration of surgery, blood loss, and transfusion requirements. The primary reason for their exclusion is the retrospective design using MIMIC-IV, which is not optimized for capturing detailed intraoperative management data. While this constraint limits the granularity of our predictions, our model offers practical value by utilizing variables that are readily available at hospital admission, enabling rapid risk assessment before surgery begins. Also our work addresses the development and internal validation phases of a prediction model using static variables at a single timepoint. Future studies leveraging surgical-specific databases included dynamic or time-series data, additional physiological, imaging, as well as advanced deep learning approaches, will be valuable for developing more comprehensive perioperative prognostic models. Third, our model predicts in-hospital mortality only and does not evaluate longer-term outcomes such as 30-day, 90-day, or 1-year mortality, as the MIMIC-IV database does not contain systematic longitudinal follow-up data beyond hospital discharge. While in-hospital mortality serves as an important endpoint for acute decision-making support during hospitalization, it may not fully capture the long-term prognostic significance of postoperative hypoxemia. Fourth, our web-based tool has demonstrated promising performance metrics in retrospective validation. We recognize that the real-world clinical impact remains to be established. Prospective implementation studies are essential to determine whether integrating the model into perioperative workflows improves decision-making, resource allocation, or patient outcomes. Finally, although we utilized the MIMIC-IV dataset for validation, future work should focus on prospective multicenter studies that incorporate extended follow-up periods and external validation across diverse healthcare systems to confirm the model’s robustness and broader applicability.

## 5. Conclusions

We developed and validated an ML model that effectively predicts in-hospital mortality risk in elderly patients who develop postoperative hypoxemia following non-cardiac surgery. By incorporating SHAP analysis, we enhanced the model’s interpretability and clinical relevance. This tool may aid in early identification and intervention for high-risk patients, potentially improving outcomes in this vulnerable population.

## Figures and Tables

**Table 1:** Baseline characteristics of the elderly patients with postoperative hypoxemia following non-cardiac surgery.

Variables	Train Data		*p*	Test Data		*p*
	Survival (*n* = 2947)	Death (*n* = 1286)		Survival (*n* = 1264)	Death (*n* = 552)	
PaO_2_/FiO_2_	160.00 (102.00, 220.00)	119.00 (77.00, 186.00)	<0.001	157.50 (100.00, 220.00)	132.00 (79.00, 201.75)	<0.001
Age (years)	76.08 (70.03, 82.72)	78.71 (72.28, 84.60)	<0.001	76.40 (70.42, 83.18)	77.96 (70.58, 85.20)	0.004
Ethnicity, *n* (%)			<0.001			<0.001
Black	204 (6.92)	82 (6.38)		95 (7.52)	39 (7.07)	
Other	667 (22.63)	393 (30.56)		298 (23.58)	200 (36.23)	
White	2076 (70.44)	811 (63.06)		871 (68.91)	313 (56.70)	
Gender, *n* (%)			0.995			0.969
Female	1373 (46.59)	599 (46.58)		576 (45.57)	251 (45.47)	
Male	1574 (53.41)	687 (53.42)		688 (54.43)	301 (54.53)	
Weight (kg)	79.33 (66.40, 94.97)	76.70 (64.03, 90.68)	<0.001	79.00 (66.64, 94.10)	74.40 (62.13, 87.30)	<0.001
Heart rate (min^−1^)	86.00 (73.00, 100.00)	92.00 (78.00, 107.00)	<0.001	85.00 (73.00, 99.25)	90.00 (76.00, 106.00)	<0.001
Sbp (mmHg)	126.00 (109.00, 144.00)	117.00 (101.00, 138.75)	<0.001	125.00 (108.75, 144.00)	121.00 (104.00, 139.00)	0.002
Dbp (mmHg)	65.00 (54.00, 76.00)	63.00 (52.00, 76.00)	0.010	65.00 (54.00, 77.00)	64.00 (53.00, 78.00)	0.553
Respiratory rate (min^−1^)	19.00 (16.00, 23.00)	21.00 (17.00, 25.00)	<0.001	19.00 (16.00, 24.00)	20.00 (17.00, 25.00)	<0.001
Temperature (°C)	36.72 (36.33, 37.11)	36.61 (36.02, 37.06)	<0.001	36.72 (36.33, 37.11)	36.56 (36.00, 36.95)	<0.001
Anion gap (mEq/L)	14.00 (12.00, 17.00)	16.00 (14.00, 20.00)	<0.001	14.00 (12.00, 17.00)	16.00 (13.00, 20.00)	<0.001
Bicarbonate (mmol/L)	23.00 (20.00, 26.00)	21.00 (17.00, 25.00)	<0.001	23.00 (20.00, 26.00)	21.00 (18.00, 25.00)	<0.001
BUN (mg/dL)	22.00 (16.00, 35.00)	31.00 (21.00, 50.00)	<0.001	23.00 (16.00, 34.00)	27.50 (19.00, 44.00)	<0.001
Calcium (mg/dL)	8.20 (7.70, 8.70)	8.10 (7.50, 8.70)	0.005	8.20 (7.70, 8.70)	8.30 (7.70, 8.80)	0.380
Chloride (mEq/L)	104.00 (100.00, 108.00)	104.00 (99.00, 108.00)	0.311	105.00 (101.00, 108.25)	104.00 (99.00, 108.00)	<0.001
Glucose (mg/dL)	141.00 (114.00, 180.00)	146.00 (113.25, 194.75)	0.040	139.50 (114.00, 179.00)	148.00 (115.00, 191.00)	0.110
Sodium (mEq/L)	139.00 (136.00, 141.00)	139.00 (136.00, 142.00)	0.009	139.00 (137.00, 142.00)	139.00 (135.00, 142.00)	0.072
Potassium (mEq/L)	4.10 (3.70, 4.60)	4.20 (3.80, 4.80)	<0.001	4.10 (3.70, 4.60)	4.25 (3.80, 4.80)	<0.001
ALP (IU/L)	74.00 (56.00, 108.00)	85.00 (62.00, 127.00)	<0.001	74.00 (55.00, 106.00)	89.00 (62.00, 130.25)	<0.001
AST (IU/L)	51.00 (28.00, 236.00)	75.50 (36.00, 399.75)	<0.001	54.00 (28.00, 236.00)	68.00 (32.00, 280.75)	0.021
PT (s)	13.90 (12.50, 16.20)	15.40 (13.20, 20.10)	<0.001	13.90 (12.50, 16.20)	14.75 (13.00, 18.90)	<0.001
APTT (s)	30.40 (27.00, 37.00)	33.95 (28.22, 47.10)	<0.001	30.90 (27.10, 37.70)	32.70 (28.00, 42.12)	<0.001
Hb (g/dL)	10.80 (9.40, 12.10)	10.60 (9.10, 12.28)	0.059	10.80 (9.40, 12.30)	10.80 (9.30, 12.22)	0.476
WBC (×10^9^/L)	11.30 (8.20, 15.80)	12.80 (8.70, 18.50)	<0.001	11.60 (8.30, 15.50)	12.95 (9.67, 18.40)	<0.001
LAC (mmol/L)	1.80 (1.20, 3.30)	2.30 (1.40, 4.50)	<0.001	1.80 (1.20, 3.10)	2.10 (1.30, 4.43)	<0.001
Mg (mg/dL)	1.90 (1.70, 2.10)	2.00 (1.70, 2.30)	<0.001	1.90 (1.70, 2.10)	2.00 (1.80, 2.20)	<0.001
Creatinine (mg/dL)	1.10 (0.80, 1.60)	1.40 (0.90, 2.20)	<0.001	1.00 (0.80, 1.50)	1.20 (0.90, 2.00)	<0.001
Charlson comorbidity index	7.00 (5.00, 8.00)	7.00 (6.00, 9.00)	<0.001	7.00 (5.00, 8.00)	7.00 (6.00, 9.00)	<0.001
Myocardial infarct, *n* (%)			<0.001			0.037
No	2393 (81.20)	954 (74.18)		1010 (79.91)	417 (75.54)	
Yes	554 (18.80)	332 (25.82)		254 (20.09)	135 (24.46)	
Congestive heart failure, *n* (%)			0.233			0.289
No	1849 (62.74)	782 (60.81)		764 (60.44)	319 (57.79)	
Yes	1098 (37.26)	504 (39.19)		500 (39.56)	233 (42.21)	
Peripheral vascular disease, *n* (%)			0.009			0.049
No	2412 (81.85)	1095 (85.15)		1038 (82.12)	474 (85.87)	
Yes	535 (18.15)	191 (14.85)		226 (17.88)	78 (14.13)	
Cerebrovascular Disease, *n* (%)			<0.001			<0.001
No	2451 (83.17)	999 (77.68)		1073 (84.89)	421 (76.27)	
Yes	496 (16.83)	287 (22.32)		191 (15.11)	131 (23.73)	
Dementia, *n* (%)			0.012			0.877
No	2798 (94.94)	1196 (93.00)		1200 (94.94)	525 (95.11)	
Yes	149 (5.06)	90 (7.00)		64 (5.06)	27 (4.89)	
Chronic pulmonary disease, *n* (%)			0.004			0.001
No	1855 (62.95)	868 (67.50)		790 (62.50)	388 (70.29)	
Yes	1092 (37.05)	418 (32.50)		474 (37.50)	164 (29.71)	
Rheumatic disease, *n* (%)			0.953			0.828
No	2813 (95.45)	1227 (95.41)		1223 (96.76)	533 (96.56)	
Yes	134 (4.55)	59 (4.59)		41 (3.24)	19 (3.44)	
Peptic ulcer disease, *n* (%)			0.875			0.468
No	2841 (96.40)	1241 (96.50)		1224 (96.84)	538 (97.46)	
Yes	106 (3.60)	45 (3.50)		40 (3.16)	14 (2.54)	
Mild liver disease, *n* (%)			<0.001			0.002
No	2706 (91.82)	1079 (83.90)		1165 (92.17)	484 (87.68)	
Yes	241 (8.18)	207 (16.10)		99 (7.83)	68 (12.32)	
Diabetes without CC, *n* (%)			0.981			0.485
No	2176 (73.84)	950 (73.87)		956 (75.63)	409 (74.09)	
Yes	771 (26.16)	336 (26.13)		308 (24.37)	143 (25.91)	
Diabetes with CC, *n* (%)			0.999			0.796
No	2688 (91.21)	1173 (91.21)		1147 (90.74)	503 (91.12)	
Yes	259 (8.79)	113 (8.79)		117 (9.26)	49 (8.88)	
Paraplegia, *n* (%)			0.001			0.001
No	2792 (94.74)	1185 (92.15)		1207 (95.49)	506 (91.67)	
Yes	155 (5.26)	101 (7.85)		57 (4.51)	46 (8.33)	
Renal disease, *n* (%)			0.027			0.380
No	2206 (74.86)	921 (71.62)		952 (75.32)	405 (73.37)	
Yes	741 (25.14)	365 (28.38)		312 (24.68)	147 (26.63)	
Malignant cancer, *n* (%)			0.615			0.260
No	2441 (82.83)	1057 (82.19)		1049 (82.99)	446 (80.80)	
Yes	506 (17.17)	229 (17.81)		215 (17.01)	106 (19.20)	
Severe liver disease, *n* (%)			<0.001			0.078
No	2870 (97.39)	1205 (93.70)		1231 (97.39)	529 (95.83)	
Yes	77 (2.61)	81 (6.30)		33 (2.61)	23 (4.17)	
Metastatic solid tumor, *n* (%)			<0.001			<0.001
No	2766 (93.86)	1163 (90.44)		1192 (94.30)	492 (89.13)	
Yes	181 (6.14)	123 (9.56)		72 (5.70)	60 (10.87)	
Hyperlipidmia, *n* (%)			<0.001			<0.001
No	1383 (46.93)	780 (60.65)		576 (45.57)	323 (58.51)	
Yes	1564 (53.07)	506 (39.35)		688 (54.43)	229 (41.49)	
IE, *n* (%)			0.929			0.306
No	2914 (98.88)	1272 (98.91)		1252 (99.05)	550 (99.64)	
Yes	33 (1.12)	14 (1.09)		12 (0.95)	2 (0.36)	
PAH, *n* (%)			0.532			0.238
No	2777 (94.23)	1218 (94.71)		1192 (94.30)	528 (95.65)	
Yes	170 (5.77)	68 (5.29)		72 (5.70)	24 (4.35)	
HTN, *n* (%)			<0.001			<0.001
No	1094 (37.12)	615 (47.82)		452 (35.76)	257 (46.56)	
Yes	1853 (62.88)	671 (52.18)		812 (64.24)	295 (53.44)	
AF, *n* (%)			0.971			0.068
No	1835 (62.27)	800 (62.21)		772 (61.08)	362 (65.58)	
Yes	1112 (37.73)	486 (37.79)		492 (38.92)	190 (34.42)	
AIDS, *n* (%)			1.000			0.757
No	2943 (99.86)	1284 (99.84)		1262 (99.84)	550 (99.64)	
Yes	4 (0.14)	2 (0.16)		2 (0.16)	2 (0.36)	
Vasoactive drugs, *n* (%)			<0.001			<0.001
No	1682 (57.07)	476 (37.01)		731 (57.83)	204 (36.96)	
Yes	1265 (42.93)	810 (62.99)		533 (42.17)	348 (63.04)	
Dopamine, *n* (%)			<0.001			<0.001
No	2793 (94.77)	1113 (86.55)		1201 (95.02)	488 (88.41)	
Yes	154 (5.23)	173 (13.45)		63 (4.98)	64 (11.59)	
Dobutamine, *n* (%)			<0.001			<0.001
No	2885 (97.90)	1204 (93.62)		1247 (98.66)	512 (92.75)	
Yes	62 (2.10)	82 (6.38)		17 (1.34)	40 (7.25)	
Epinephrine, *n* (%)			<0.001			<0.001
No	2830 (96.03)	1097 (85.30)		1235 (97.71)	479 (86.78)	
Yes	117 (3.97)	189 (14.70)		29 (2.29)	73 (13.22)	
Phenylephrine, *n* (%)			<0.001			<0.001
No	2073 (70.34)	734 (57.08)		882 (69.78)	315 (57.07)	
Yes	874 (29.66)	552 (42.92)		382 (30.22)	237 (42.93)	
Norepinephrine, *n* (%)			<0.001			<0.001
No	2002 (67.93)	432 (33.59)		858 (67.88)	206 (37.32)	
Yes	945 (32.07)	854 (66.41)		406 (32.12)	346 (62.68)	
Vasopressin, *n* (%)			<0.001			<0.001
No	2667 (90.50)	799 (62.13)		1165 (92.17)	377 (68.30)	
Yes	280 (9.50)	487 (37.87)		99 (7.83)	175 (31.70)	
CRRT, *n* (%)			<0.001			<0.001
No	2867 (97.29)	1213 (94.32)		1242 (98.26)	526 (95.29)	
Yes	80 (2.71)	73 (5.68)		22 (1.74)	26 (4.71)	

Continuous values were presented as median [interquartile range]. Categorical values were presented as number (percentage). Sbp: systolic blood pressure; Dbp: diastolic blood pressure; BUN: blood urea nitrogen; ALP: alkaline phosphatase; AST: aspartate aminotransferase; PT: prothrombin time; APTT: activated partial thromboplastin time; Hb: hemoglobin; WBC: white blood cell; LAC:lactic acid; Mg: magnesium; CC: complication or comorbidity; IE: infective endocarditis; PAH: pulmonary artery hypertension; HTN: hypertension; AF: atrial fibrillation; AIDS: acequired immune deficiency syndrome; CRRT: continuous renal replacement therapy.

**Table 2:** Six models performance metrics.

Model	Accuracy	F1 Score	Sensitivity	Specificity	PPV	NPV	AUC
DT	0.71	0.52	0.50	0.80	0.53	0.79	0.72
LightGBM	0.66	0.57	0.76	0.61	0.46	0.85	0.77
MLP	0.70	0.57	0.65	0.73	0.51	0.83	0.77
RF	0.69	0.58	0.71	0.68	0.49	0.84	0.78
RSVM	0.70	0.58	0.69	0.70	0.50	0.84	0.78
XGBoost	0.77	0.53	0.43	0.92	0.69	0.79	0.79

DT: Decision Tree; LightGBM: Light Gradient Boosting Machine; MLP: Multi-layer Perceptron; RF: Random Forest; RSVM: Radial Support Vector Machine; XGBoost: Extreme Gradient Boosting; PPV: Positive Predictive Value; NPV: Negative Predictive Value; AUC: Area Under the Receiver Operating Characteristic Curve.

## Data Availability

The original contributions presented in this study are included in the body of the article. Further inquiries can be directed to the corresponding author.
